# Correlates for Suicidality Among At-risk Youth Receiving Community-Based Mental Health Services

**DOI:** 10.1007/s10597-022-01011-y

**Published:** 2022-08-01

**Authors:** Karen L. Celedonia, Max Karukivi, Anne Abio, Michael W. Valenti, Michael Lowery Wilson

**Affiliations:** 1 Injury Epidemiology and Prevention (IEP) Research Group, Turku Brain Injury Centre, Turku University Hospital and University of Turku, Turku, Finland; 2 Department of Adolescent Psychiatry, Turku University Hospital and University of Turku, Turku, Finland; 3 Heidelberg Institute of Global Health, Heidelberg University, Heidelberg, Germany; 4 Social Research and Innovation Center, Pressley Ridge, Pittsburgh, PA USA

**Keywords:** Adolescent suicide, Suicidality correlates, “At-risk” populations, Community-based samples

## Abstract

“At-risk” adolescents are at high risk of unsuccessfully transitioning into adulthood and are also at elevated risk for suicidal behavior. Though much research has been conducted on risk factors for suicidality among the general adolescent population, research on suicidality among “at-risk” adolescents is lacking. This is a notable gap in the literature given that “at-risk” adolescents may be three times more likely to exhibit suicidality. The present study addressed this research gap by examining correlates for suicidality among “at-risk” adolescents receiving mental health services in the community. Using Electronic Health Record (EHR) data, risk factors for suicidality were analyzed at the bivariate and multivariate levels. Sexual abuse was a significant predictor of suicidality, as well as impulsivity for suicide attempt only. These findings may serve as useful adjuncts in the design of suicidality-screening tools and follow-up practices within the context of community-based mental health organizations which target at-risk adolescents.

## Introduction

Suicide is the second-leading cause of death among adolescents in the United States (Hedegaard et al., 2020). In order to prevent death by suicide from occurring, it is necessary to understand risk factors for suicidality. Suicidality is a continuum that includes suicidal ideation, suicide attempt, and death by suicide. Among the general population of adolescents in the United States, prevalence of suicidal ideation is around 15%, and prevalence of suicide attempt is around 7% (*Epidemiology of youth suicide and suicidal behavior: Current Opinion in Pediatrics*, 2021). More recent research on lifetime prevalence of suicidality among adolescents in the United States indicates a prevalence of 12% for suicidal ideation and 4% for suicide attempt (Nock et al., 2013).

Research on adolescent suicidality has identified numerous risk factors that are repeatedly found to be associated with increased risk of suicidality. Gender seems to be a clear risk factor, with girls having elevated odds of suicidal ideation and suicide attempt (Nock et al., 2013). In the United States, race may be a risk factor, with non-Hispanic White adolescents having higher odds of suicidal ideation and suicide attempt (Nock et al., 2013). Presence of a psychiatric disorder, especially depression, is also significantly associated with increased risk of suicidality (Miller et al., 2013). Other well-established risk factors for suicidality include bullying victimization, child maltreatment (i.e. physical abuse, sexual abuse, and neglect), exposure to family discord (i.e. domestic violence), impulsivity (Miller et al., 2013; Strohacker et al., 2019), and trait anger (Daniel et al., 2009; Lehnert et al., 1994).

Though much research on risk factors for adolescent suicide and suicidality exists, this body of research has been conducted primarily using community-based samples from high schools or universities, clinical samples from inpatient psychiatric hospitals or university clinics, or population-based samples. Studies that examine suicidality using targeted samples of “at-risk” adolescents receiving mental health services in the community setting are few (McBride et al., 2017). “At-risk” adolescents are defined as individuals who have been or continue to be exposed to a host of negative environmental and social factors, otherwise known as Adverse Childhood Experiences (ACEs) (Fernandes-Alcantara, 2014), and are therefore “at-risk” of not transitioning successfully to adulthood because of these early challenges in their lives (What is at-risk youth?—Definition & statistics, 2016). Most “at-risk” adolescents are emancipating foster youth, runaway and homeless youth, or youth involved in the juvenile justice system (Fernandes-Alcantara, 2014).

ACEs are stressful or traumatic events that occur between birth and 18 years of age, and include homelessness/transient living environment; stressful family environments (i.e. domestic violence); lack of social or emotional supports; various forms of abuse (physical, sexual, and verbal); neglect (physical and emotional); bullying victimization; and loss of a parent or loved one (Felitti et al., 1998; Fernandes-Alcantara, 2014); Centers for Disease Control & Prevention, 2019). Exposure to these negative environmental and social factors may contribute to the development of behavioral and mental health disorders (McLaughlin et al., 2012; Schmidt, 2007).

A review of the literature on suicidality among “at-risk” adolescents revealed that the extant literature on the topic is lacking in both quantity and quality. As such, there is a need for more research on suicidality among this sub-population of adolescents. It cannot be assumed that the findings from research on suicidality among the general adolescent population are generalizable to this niche population. The life experiences of “at-risk” adolescents are not comparable to the general adolescent population, and consequently, the phenomenon of suicidality among “at-risk” adolescents requires special examination. Exposure to acute trauma during an individual’s formative years—like that experienced by “at-risk” adolescents—alters brain development and physiological composition in such a manner that predisposes one to maladaptive reactions to stress (Belsky & de Haan, 2011; Grasso, et al., 2021).

Consequently, these neurological and physiological alterations may render “at-risk” adolescents more prone to suicidal behavior. Many ACEs are associated with increased risk of suicidality and are the very same risk factors that have been extensively researched by suicidologists. For example, history of sexual abuse and physical abuse are associated with an increased risk of suicidal ideation and behavior among adolescents (Fergusson et al., 2008). Studies suggest that cumulative trauma—experiencing more than one traumatic event—may result in an even greater increase in suicidality risk (Johnson, 2017). Dube et al. (2001) found that each individual ACE item increases the odds of suicide attempt by two- to five-times, and suicidal behavior increases exponentially for each additional ACE that is endorsed (Dube et al., 2001). Furthermore, adolescents who have experienced traumatic events like ACEs are three-times more likely to be suicidal than adolescents who have not experienced traumatic events (Brown et al., 1999). The compounding effect of cumulative ACEs on suicidality risk is particularly concerning for “at-risk” adolescents given that research suggests this sub-population of adolescents is at increased risk for cumulative childhood adversities (Turney & Wildeman, 2017).

Though ACEs increase suicidality risk, there are protective factors that can mitigate the deleterious effects of ACEs on the developing brain. Some of these protective factors include family communication and school connection (Lensch et al., 2021). However, most “at-risk” adolescents do not have strong family ties and may move from school district to school district due to unstable living environments or struggle with truancy. Not having access to these protective factors further distinguishes “at-risk” adolescents from the general adolescent population in regards to increased susceptibility to exhibiting suicidal behavior.

The aim of the present study was to examine the prevalence and correlates of suicidality among “at-risk” adolescents receiving behavioral and mental health services in the community. Risk factors well-established in the literature on adolescent suicidality were examined to determine if these same risk factors were associated with suicidality among “at-risk” adolescents. The risk factors examined were gender, race, psychiatric diagnosis, sexual abuse, physical abuse, exposure to domestic violence, bullying victimization, impulsivity, loss of loved one, and anger. The results from the study can be used to inform clinical practice at community-based organizations that serve this sub-population of adolescents. An awareness of which risk factors are most prominent and which factors are significantly associated with suicidality might help providers prevent suicidal behavior and could support more informed treatment decisions. Results from this analysis may also provide a blueprint for future research among this population.

## Methods

### Setting

This study was conducted at a multi-state child welfare organization in the United States. The organization delivers a wide array of behavioral and mental health services to youth, families, and adults in local communities. The organization offers services through five distinct service lines, all of which are delivered in the community: residential services, community-based services (i.e. crisis response, in-home family-based services), outpatient mental health services, treatment foster care and adoption, and special education. Individuals are either self-referred to services or referred by external entities, such as child protection services or the juvenile justice department.

### Data

Study data were derived from the clinical electronic health records (EHR) of the organization. Data extracted for analysis were de-identified. When using claims data or other secondary datasets (i.e. EHR data), the variables that are selected for analysis are often limited to the available data (Robst et al., 2011). The independent variables that were examined in this study included: gender, race, psychiatric diagnosis, history of physical abuse, history of sexual abuse, bullying victimization, domestic violence exposure, impulsivity, anger, and death of a loved one/someone important in the adolescent’s life. Approval for the research was granted by the organization’s internal research review committee (Celedonia et al., 2020).

### Sample

The initial sample included 1,236 unique individuals between the ages of 13 and 18 who were screened by trained clinicians for suicidal behavior at program intake between July 1, 2019 and June 30, 2020. This time frame was selected to include the entire fiscal year of 2020. The clinicians who conducted the screening were employees of the organization and had Bachelor’s or Master’s degrees in psychology, social work, or other behavioral-health related majors. They also had completed a mandatory suicidality assessment training in which the screener was reviewed and instruction for administering the screener was provided. Clinicians are required to screen youth for suicidality at intake per organization standard operating procedures. In cases of youth with multiple screeners, the screeners completed at the earliest date were kept, and all those thereafter were excluded from the sample, so as to ensure that only screeners completed at intake were included in the analysis. Youth with no completed trauma screen in addition to the suicidal behavior screen were excluded from the sample (n = 627), as the trauma screen was used to extract independent variables being tested in the study. A more detailed description of the trauma screen is provided in the section on Trauma and Adverse Experiences. Youth with an outdated version of the trauma screen were also excluded (n = 297), as well as youth with the caregiver version of the trauma screen (n = 13) and youth with blank trauma screens (n = 10). The final sample used for analysis included 289 youth 13–18 years old.

### Variables and Measurement

#### Dependent Variable: Suicidal Behavior

To assess suicidal behavior, the Columbia Suicide Severity Rating Scale (C-SSRS)-Screener was used. The C-SSRS is a standardized measure of suicidal ideation and behavior with robust psychometrics (Posner et al., 2011). It is widely used in research and clinical practice to assess suicidality among youth and adults. The C-SSRS-Screener is a 6-item suicidality screen that assesses suicidal ideation, planning, and attempts. For the purposes of this study, to screen positive for suicidality youth had to endorse “Yes” to Question 1 (“Have you wished you were dead or wished you could go to sleep and not wake up?”), Question 2 (“Have you actually had any thoughts of killing yourself?”), or Question 6 (“Have you ever done anything, started to do anything, or prepared to do anything to end your life?”) on the C-SSRS. Two dichotomized dependent variables of suicidal behavior were created: 1) 1 = Suicidal Behavior Present (Yes to Question 1, Question 2, OR Question 6) and 0 = No Suicidal Behavior Present, and 2) 1 = Suicidal Behavior Present (Yes to Question 1, Question 2, AND Question 6) and 0 = No Suicidal Behavior Present. Suicidal ideation and suicide attempt were also analyzed separately. For suicidal ideation, the youth had to endorse “Yes” to Question 1 or Question 2. For suicide attempt, the youth had to only endorse Question 6. The suicide variables were not exclusive, meaning one participant could be included in multiple categories.

#### Independent Variables: Gender and Race

The variables of gender and race were extracted from the organization’s EHR. These demographic variables are a combination of self-report on the organization’s intake documentation and third-party reporting if the organization does not collect the information. On the organization’s intake form, there are options for individuals to select a gender other than male or female (i.e. Transgender-presents as Male or Transgender-presents as Female). For analysis purposes, race was coded into a dichotomized variable consisting of White and non-White. This was done to create more comparable sample sizes, as some of the races, such as Asian, had only one individual identifying as such. Detailed demographic data are provided on all races included in the study in the Results section.

#### Independent Variable: Psychiatric Diagnosis

Psychiatric diagnosis was extracted from the organization’s EHR. Clinicians used ICD-10 codes and DSM-5 categories to identify diagnoses. Both of these diagnoses are entered into the EHR. For the purpose of this study, the DSM-5 diagnoses were used. Diagnoses were initially coded according to DSM-5 categories, and then, for analysis purposes, diagnoses were coded into the following categories: No psychiatric diagnosis, Internalizing Disorders (Depressive Disorders; Anxiety Disorders; Bipolar Disorder, Autism, Personality Disorders, and Trauma and Stress Related Disorders) and Externalizing Disorders (ADHD and Disruptive, Impulse-Control, and Conduct Disorders). Only primary diagnoses were included in the analysis. Another member of the research team reviewed the diagnostic categories for consensus and approval before the analysis was conducted. Detailed data on the distribution of the individual disorders are provided in the Results section.

#### Independent Variables: Trauma/Adverse Experiences

To assess various trauma or adverse experiences variables typically associated with suicidal behavior, the Children and Adolescent Trauma Screen (CATS) was used. The CATS is a standardized measure with strong psychometrics that is used in the clinical and research setting to assess trauma experienced by children and adolescents (Sachser et al., 2017). For this study, the Youth Report version was used. Items were extracted from the CATS based on their relevance to suicidality as identified in the extant literature. These items included history of physical abuse (Part 1, Question 3: “Threatened, hit, or hurt badly within the family”), history of sexual abuse (Part 1, Question 8: “Someone doing sexual things to you or making you do sexual things to them when you couldn’t say no. Or when you were forced or pressured”), bullying victimization (Part 1, Question 10: “Someone bullying you in person. Saying very mean things that scare you” or Part 1, Question 11: “Someone bullying you online. Saying very mean things that scare you”), exposure to domestic violence (Part 1, Question 6: “Seeing someone in the family threatened, hit, or hurt badly”), impulsivity (Part 2, Question 16: “Doing unsafe things”), loss of a loved one/someone important in the child’s life (Part 1, Question 12: “Someone close to you dying suddenly or violently”), and anger (Part 2, Question 15: “Feeling mad. Having fits of anger and taking it out on others”). Questions from Part 1 are scored on a Yes/No scale. Questions from Part 2 are scored on 0–3 Likert scale, with 0 = Never and 3 = Almost always. For Questions from Part 1, presence of the variable was coded as 1 = present (Yes) and 0 = not present (No). For questions from Part 2, presence of the variable was coded as 1 = present (Once in a while, Half the time, or Almost always) and 0 = not present (Never). Table 1 provides a summary of how independent variables were derived from the CATS.

**Table 1:** Independent variable derivation from the Child and Adolescent Trauma Screen (CATS)

Survey question	Coding	Variable
Threatened, hit, or hurt badly within the family	Yes (1); No (0)	Physical abuse
Someone doing sexual things to you or making you do sexual things to them when you couldn’t say no. Or when you were forced or pressured	Yes (1); No (0)	Sexual abuse
Someone bullying you in person. Saying very mean things that scare you OR Someone bullying you online. Saying very mean things that scare you	Yes (1); No (0)	Bullying Victimization
Seeing someone in the family threatened, hit, or hurt badly	Yes (1); No (0)	Domestic violence
Doing unsafe things	Once in a while, Half the time, or Almost always (1); Never (0)	Impulsivity
Someone close to you dying suddenly or violently	Yes (1); No (0)	Loss of loved one
Feeling mad. Having fits of anger and taking it out on others	Once in a while, Half the time, or Almost always (1); Never (0)	Anger

### Statistical Analysis

The distribution of the independent variables within the dichotomized suicidal behavior variables, suicidal ideation variable, and suicide attempt variable were examined first. Differences between suicidality among the variables was assessed for statistical significance using Pearson’s chi-square test for categorical variables. For independent variables found to be statistically significant at the bivariate level (*P* < .05), a logistic regression model that adjusted for these variables was developed and tested. The bivariate analysis was done using SPSS 25, and the logistic regression was done using R Studio 3.5.3. Multicollinearity was tested on all four models using the variance inflation factor (VIF), and all VIF values were around 1, indicating that multicollinearity was not present.

## Results

### Sample Demographics

The average age of the youth was 15.6 years. Fifty-two percent of youth were female, 48% were male, and 0% were transgender. Two-thirds (66%) of the youth were Caucasian, 22% were Black or African American, 10% were Bi- or Multi-racial, and 2% were Hispanic/Latino or Asian. One third (33%) of youth were receiving community-based services, 28% were receiving outpatient mental health services, 27% were receiving residential services, 11% were receiving treatment foster care and adoption services, and 1% were receiving special education services. One fifth (n = 57; 20%) of youth were diagnosed with Trauma and Stressor Related Disorders as their primary diagnoses at intake. Other common primary diagnoses included ADHD (n = 47; 16%), Disruptive, Impulse-Control, and Conduct Disorders (n = 42; 15%), and Depressive Disorders (n = 39; 13%). Almost forty-percent (n = 110; 38%) of adolescents screened positive for suicidal behavior (suicidal ideation and/or suicide attempt) at program intake. Almost a third screened positive for suicidal ideation (n = 94; 32.5%), and one quarter screened positive for suicide attempt (n = 67; 25.5%). See Table 2 for a detailed summary of the sample demographics.

**Table 2:** Sample demographics

Demographic categories	N	Percent (%)	Mean
Age	289		15.6 years
Gender			
Male	139	48	
Female	150	52	
Transgender	0	0	
Race			
Caucasian	190	66	
Black or African American	63	22	
Bi- or Multi-racial	28	10	
Hispanic/Latino or Asian	5	2	
Service line			
Foster care and adoption	30	11	
Outpatient	73	28	
Community-based	91	33	
Special education	2	1	
Residential	67	27	
Primary diagnosis			
Trauma and stressor-related disorders	57	20	
ADHD	47	16	
Disruptive, impulse- control, and conduct disorders	42	15	
Depressive disorders	39	13	
Anxiety disorders	13	5	
Autism	2	2	
Bipolar disorder	3	1	
Personality disorders	1	< 1	
No mental health diagnosis	82	28	
Suicidality			
Suicidal behavior (suicidal ideation and/or suicide attempt)	110	38	
Suicidal ideation	94	33	
Suicide attempt	67	26	

### Bivariate Analysis

All independent variables were significant at the bivariate level for the suicidal behavior variable of ideation or attempt, except race (*P* = .534). All independent variables were significant at the bivariate level for the suicidal behavior variable of ideation and attempt, except race (*P* = .943) and loss of loved one (*P* = .080). All independent variables were significant at the bivariate level for suicidal ideation, except race (*P* = .457). All independent variables were significant at the bivariate level for suicide attempt, except race (*P* = .900) and loss of loved one (*P* = .079). Sexual abuse was most significantly associated with all of the suicidality variables with *P*-values of .000 for all four distinct variables. Table 3 provides detailed results, including percentages and Pearson chi-square values.

**Table 3:** Results from bivariate analyses: suicidal behavior among an at risk population of adolescents receiving community-based mental health services

Variable	SB (SI OR SA)			SB (SI AN D SA)			SI			SA		
	SB (%)	No SB (%)	*P*	SB (%)	No SB (%)	*P*	SI (%)	No Sl (%)	*P*	SA (%)	No SA (%)	*P*
Gender (male)	36.4	55.3	.002	33.3	51.3	.020	38.3	52.8	.021	31.3	51.5	.004
Race (white)	64.2	67.8	.534	66.0	66.5	.943	63.4	67.9	.457	66.7	65.8	.900
Diagnosis (internalizing)	56.4	31.3	.000	58.8	37.0	.014	55.3	33.8	.002	59.7	34.7	.002
Physical abuse	48.2	29.1	.001	62.7	30.7	.000	48.9	30.3	.002	58.2	28.6	.000
Sexual abuse	41.8	15.3	.000	56.9	18.6	.000	43.6	16.6	.000	50.7	16.5	.000
Bullying victimization	51.4	32.0	.001	54.9	36.0	.012	52.1	33.2	.002	53.0	33.8	.006
Domestic violence	53.6	35.2	.002	64.7	37.4	.000	53.2	36.9	.009	62.7	35.2	.000
Loss of loved one	58.7	41.0	.004	58.8	45.3	.080	60.6	41.5	.002	56.1	43.6	.079
Impulsivity	44.9	28.7	.006	56.9	30.0	.000	45.7	29.6	.009	53.0	27.4	.000
Anger	66.4	51.8	.018	76.5	53.2	.002	67.4	52.5	.019	72.7	50.8	.002

*SB* suicidal behavior, *SI* suicide ideation *SA* suicide attempt, *P* p-value

### Multivariate Analysis

All independent variables found to be significantly associated with suicidal behavior, suicidal ideation, and suicide attempt at the bivariate level were analyzed using a binomial logistic regression to test for predictors. Four models were tested: (1) suicidal ideation and suicide attempt, (2) suicidal ideation or suicide attempt, (3) suicidal ideation, and( 4) suicide attempt. History of sexual abuse was found to be a significant predictor across all four models (OR 2.52, CI 1.33–4.78; OR 3.12, CI 1.48–6.56; OR 2.79, CI 1.47–5.30; and OR 2.43, CI 1.19–4.96, respectively). Impulsivity was a significant predictor only in the suicide attempt model (OR 2.16; CI 1.05–4.43). No other independent variables were found to be significant predictors in any of the models. See Table 4 for details of the results.

**Table 4:** Results from multivariate analyses: suicidal behavior among an at risk population of adolescents receiving community-based mental health services

Variable	SB(SI OR SA)			SB(SI AND SA)			SI			SA		
	OR	95% CI	*P*	OR	95% CI	*P*	OR	95% CI	*P*	OR	95% CI	*P*
Gender (male)	1.66	0.95–2.90	.073	1.71	0.81–3.62	.155	1.36	0.76–2.41	.297	1.92	0.97–3.80	.061
Diagnosis (internalizing)	0.95	0.67–1.36	.788	1.05	0.65–1.70	.844	0.97	0.67–1.40	.862	1.09	0.71–1.68	.691
Physical abuse	1.30	0.71–2.37	.394	2.01	0.95–4.26	.067	1.29	0.70–2.39	.418	1.82	0.91–3.64	.093
Sexual abuse	2.52	1.33–4.78	.004	3.12	1.48–6.56	.003	2.79	1.47–5.30	.002	2.43	1.19–4.96	.015
Bullying victimization	1.48	0.85–2.55	.165	1.08	0.53–2.22	.827	1.45	0.82–2.55	.196	1.31	0.69–2.49	.414
Domestic violence	1.25	0.71–2.20	.445	1.62	0.78–3.35	.194	1.16	0.65–2.08	.619	1.69	0.88–3.27	.118
Loss of loved one	1.63	0.93–2.82	.085	1.17	0.57–2.40	.659	1.73	0.98–3.05	.060	n/a	n/a	n/a
Impulsivity	1.50	0.80–2.79	.208	2.01	0.93–4.34	.075	1.42	0.75–2.68	.286	2.16	1.05–4.43	.036
Anger	1.22	0.66–2.56	.256	1.76	0.75–4.10	.191	1.25	0.66–2.37	.487	1.48	0.70–3.11	.307

*SB* suicidal behavior, *SI* suicide ideation, *SA* suicide attempt *P* p-value, *OR* odds ratio, *95% CI* 95% confidence interval

## Discussion

The results from the present study provide much needed insight into the suicidality risk profile of “at-risk” adolescents. Though substantial research has been and continues to be conducted on suicidality among adolescents in general, suicidality within the sub-population of “at-risk” adolescents is under-researched, and as such, there is a dearth of high-quality research available on this population. This is an important gap in the literature to rectify considering that “at-risk” adolescents are likely to be at increased risk of death by suicide due to a threefold increase in risk of being suicidal (Brown et al., 1999).

The results from the present study support previous research on risk factors among adolescents, with most of the independent variables examined significantly associated with suicidality at the bi-variate level. Exceptions were race, which was not significantly associated with any of the four suicidality variables, and loss of loved one, which was not significantly associated with suicide attempt only. Though race has been found to be associated with suicidality, there is not a consistent trend in this association in the literature. Some studies report that non-Hispanic White adolescents are at increased risk of suicidality (Nock et al., 2013, while others report that adolescents of color are at increased risk of suicidality (Nestor et al., 2016; Lindsey et al., 2019). Since there are no consistent findings in the research on the association between race and suicidality, it is not surprising that race was not associated with suicidality in the present study. It is yet another variability in the findings on the association between race and suicidality.

As far as loss of loved one not being significantly associated with suicide attempt, one explanation for this finding could be that the treatment the adolescent was receiving at the community-based mental health organization helped the adolescent cope with the loss of their loved one, thereby attenuating any suicidal behavior that may have developed as a result of this trauma. Another explanation could be the nature of the grieving process as conceptualized by Kubler-Ross’s Five Stages of Grief (Kubler-Ross, 1969). When an individual loses a loved one, they initially go through stages of denial, anger, and bargaining before arriving at depression and then finally transforming their pain into acceptance in the final stage. Though they may ideate throughout these stages, moving through the grief process may help to prevent an individual from acting out on their suicidal thoughts.

A few other noteworthy findings also emerged from the analysis. First, the prevalence of suicidal behavior (suicidal ideation and/or suicide attempt) among this sample of “at-risk” adolescents was almost forty percent. The prevalence of suicidal ideation was 32.5%, which is double the prevalence of suicidal ideation among the general adolescent population, and the prevalence of suicide attempt was 25.5%, which is triple the prevalence of suicide attempt among the general adolescent population (Nock et al., 2013). These findings add to the evidence that “at-risk” adolescents are at increased risk of experiencing suicidality, further reiterating the need to conduct more targeted research on this sub-population to understand how their suicidality risk profile differs from that of the general adolescent population.

Perhaps most significant was the finding that only sexual abuse was a significant predictor of suicidality. Victims of sexual abuse commonly suffer from myriad challenges associated with the risk factors of suicidality. For example, some studies demonstrate that up to 75% of adolescents who experience sexual abuse receive abuse-related psychiatric diagnoses as a result (Murat et al., 2015). This is relevant, as psychiatric diagnosis is one of the primary risk factors predicting suicidal behavior (Bachmann, 2018). Other researchers have noted additional adverse effects of sexual abuse, including substance abuse problems, anger management issues, low self-esteem, sleep deprivation, increased stress, and sexualized behaviors (Malhotra & Biswas, 2006). It is possible that individuals dealing with these problems may struggle to cope with stressful life events that often predate suicidal behavior. It is not surprising, then, sexual abuse was a significant predictor of suicidality for at-risk adolescents in this sample.

What may be surprising is that none of the *other* factors were significant predictors of suicidal behavior across all multivariate statistical models. Extant research shows that some of these factors, including physical abuse and exposure to bullying, are often associated with increased risk for suicidal behavior (Alavi et al., 2015; Salzinger et al., 2007). In this very sample, all included factors excluding race and loss of loved one were significant predictors at the bivariate level. Why these factors did not remain significant predictors in multivariate analyses in this high-risk population warrants future research and exploration. One possibility is that there may be an unmeasured association between some of these factors and the type of treatment received by participants in this community sample. That is, participants may be receiving treatment for certain risk factors that may have reduced symptomology associated with elevated suicide risk.

While risk factors such as these are often associated with suicidality in high-risk populations, some factors may be more influential than others. Concerning forms of abuse and maltreatment, a review of the existing literature finds that sexual abuse has more significance for explaining suicidal behavior than either physical abuse or neglect (Miller et al., 2013). Being a victim of sexual abuse may be uniquely predictive of future suicidal behavior and warrants additional exploration in at-risk populations. Future research could examine why sexual abuse has such a significant impact on suicidal behavior, including how sexual abuse influences other risk factors not included in the analyses in this study. In the interim, we suggest that service providers routinely screen for sexual abuse independently or as part of a broader trauma screening battery. Given the potential increase in suicidal behavior risk associated with sexual abuse, identifying history and providing targeted treatment may be paramount to preventing future suicidal behavior.

Though impulsivity was found to be a significant predictor, it was only found to be such in the suicide attempt model. Theories of suicide have suggested that the progression from suicidal ideation to suicide attempt is a process that individuals go through with various steps in one’s thought process and feelings (Klonsky & May, 2015). Having impulsive tendencies may preclude an individual from the benefit of thinking through the implications of ending their life before acting on self-injurious desires. Perhaps there is something about the cognitive, ruminative aspect of ideation that mitigates the risk for suicide attempt and explains why most people who consider suicide rarely decide to act on their thoughts (Klonsky & May, 2014; Nock et al., 2008). A person who is impulsive, however, may be predisposed to skip the ideation step and go directly to a suicide attempt. Interestingly, impulsive behavior has been linked to childhood adversity: individuals who experience marked levels of childhood adversity are more likely to develop impulsive behavior (Lovallo, 2013). This link between childhood adversity and impulsivity is particularly relevant to the study population of “at-risk” adolescents. Given our findings, clinicians providing services to “at-risk” adolescents may want to focus treatment efforts on decreasing impulsive behavior as a means of preventing suicide attempts.

The present study is not without limitations, however. As mentioned previously, the independent variables studied were restricted by the data available in the organization’s EHR. As such, though the results provide a starting point for developing a suicidality risk profile for “at-risk” adolescents, it is not a complete risk profile. Future research should expand on the present study’s findings and include variables that were missing from the present study’s analysis, like sex trafficking victimization and family history of death by suicide, which are indicated in the literature as being associated with suicidality (Frey et al., 2019; Nakagawa et al., 2009). Other limitations include sample size attrition due to absence of the trauma screener in conjunction with the suicide screener and the study’s four-month overlap with the COVID-19 pandemic, which could have resulted in an increase of various trauma factors, such as physical abuse and domestic violence exposure (Lawson et al., 2020; Usher et al., 2020). Though experts warned of increased suicidal behavior as a result of the pandemic, a review of the research indicates that the pandemic may not have had the negative effect on suicidal behavior that was anticipated, with suicide rates either remaining the same or decreasing (Tandon, 2021).

Limitations aside, the present study adds to the body of literature on suicidality among “at-risk” adolescents. The results from the present study also have implications for clinical practice as well as practical utility. Given that the study was conducted using EHR data at a child welfare organization, the results will be presented to administrative and clinical leadership at the organization in order to develop an action plan based upon the results to modify and bolster the organization’s current suicidality screening protocol. With the finding that sexual abuse was the only significant predictor across all four models, it may be suggested that those adolescents who screen positive for history of sexual abuse be routinely monitored for changes in suicidality and additional safeguards to protect against suicide attempt and death by suicide are implemented for these adolescents.
